# Molecular Design of Porous Organic Polymer-Derived Carbonaceous Electrocatalysts for Pinpointing Active Sites in Oxygen Reduction Reaction

**DOI:** 10.3390/molecules28104160

**Published:** 2023-05-18

**Authors:** Xiaofeng Mou, Xiaoyu Xin, Yanli Dong, Bin Zhao, Runze Gao, Tianao Liu, Na Li, Huimin Liu, Zhichang Xiao

**Affiliations:** Department of Chemistry, College of Science, Hebei Agricultural University, Baoding 071001, China; mxf18282919668@163.com (X.M.); 13180500262@163.com (X.X.); dyl19810314@126.com (Y.D.); zhaobin@hebau.edu.cn (B.Z.); 13718206685@139.com (R.G.); kukuchanj@163.com (T.L.); miaona628510@foxmail.com (N.L.)

**Keywords:** porous organic polymers, carbocatalysts, oxygen reduction reaction, molecular level, structure-property relationship

## Abstract

The widespread application of fuel cells is hampered by the sluggish kinetics of the oxygen reduction reaction (ORR), which traditionally necessitates the use of high-cost platinum group metal catalysts. The indispensability of these metal catalysts stems from their ability to overcome kinetic barriers, but their high cost and scarcity necessitate alternative strategies. In this context, porous organic polymers (POPs), which are built up from the molecular level, are emerging as promising precursors to produce carbonaceous catalysts owning to their cost-effectiveness, high electrical conductivity, abundant active sites and extensive surface area accessibility. To enhance the intrinsic ORR activity and optimize the performance of these electrocatalysts, recognizing, designing, and increasing the density of active sites are identified as three crucial steps. These steps, which form the core of our review, serve to elucidate the link between the material structure design and ORR performance evaluation, thereby providing valuable insights for ongoing research in the field. Leveraging the precision of polymer skeletons based on molecular units, POP-derived carbonaceous catalysts provide an excellent platform for in-depth exploration of the role and working mechanism for the specific active site during the ORR process. In this review, the recent advances pertaining to the synthesis techniques and electrochemical functions of various types of active sites, pinpointed from POPs, are systematically summarized, including heteroatoms, surficial substituents and edge/defects. Notably, the structure–property relationship, between these active sites and ORR performance, are discussed and emphasized, which creates guidelines to shed light on the design of high-performance ORR electrocatalysts.

## 1. Introduction

With the ever-growing consumption of fossil fuels and the exacerbation of environmental problems, it is imperative to seek clean energy conversion/storage equipment to meet the sustainable supply of future energy consumption [1]. Proton exchange membrane fuel cells (PEMFCs), which consume hydrogen at the anode and oxygen at the cathode, make for an ideal electrochemical device because of their environmental friendliness and high energy output. However, the slow dynamics and poor stability of the cathodic oxygen reduction reaction (ORR) lead to the low efficiency and inferior performance of these PEMFC devices [2]. Therefore, it is imperative to explore high-efficiency ORR catalysts to promote the development of these clean energy conversion systems. Up to now, the platinum group metal (PGM) materials, including platinum and platinum-based catalysts, have long been regarded as the most efficient ORR electrode materials [3]. However, the high cost and scarcity of platinum, together with several other drawbacks, including the time-dependent drift, methanol crossover, and CO deactivation, have impeded their commercial application for PEMFCs. Reducing the dosage of platinum, or directly substituting a high-performance PGM-free catalyst for platinum, has become significant within the field of electrocatalytic ORR.

Since the pioneering work of Dai’s group in 2009, in which nitrogen-doped vertically aligned carbon nanotubes (VA-CNTs) were demonstrated to be superior to a platinum-based catalyst with better tolerance to the crossover effect in alkaline fuel cells [4], carbonaceous metal-free electrocatalysts stand out as a promising alternative for the ORR process, owing to their abundance on earth, low cost, structural stability during the electrochemical process, and the highly accessible active sites inside the carbonaceous skeleton. From the carbon chemistry point of view, traditional pristine carbon nanomaterials, such as fullerene, carbon nanotube, and graphene, are mainly characterized by hybridization. However, such carbon allotropes, with a sizeable basal plane, always exhibit low oxygen adsorption energy because of the stable electronic configuration of the (0001) surface orientation [5]. Worse still, the possible two-electron ORR process may compete with the target four-electron ORR process in the PEMFC. As a result, it is imperative to find a desirable carbocatalyst for the efficient and selective four-electron ORR process. To accomplish this goal, the recognition, construction and intensification of the active sites are the three key steps. Nevertheless, within the current methods [6] for fabricating carbonaceous electrocatalysts, including the biomass derivatization method, post treatment method, and chemical/physical etching method, it is always difficult to control the design of certain types of active site and can only form a combination of several kinds of active sites in the carbonaceous skeleton.

Porous organic polymers (POPs), which are based on covalent bonds, are emerging as a porous material, due to their abundant building units and flexible synthetic methods [7]. Generally speaking, polymers of intrinsic microporosity (PIMs), conjugated microporous polymers (CMPs), hyper-cross-linked polymers (HCPs), covalent triazine frameworks (CTFs), covalent organic polymers (COPs), and polycyclic aromatic hydrocarbons (PAHs) are all categorized as POPs, depending on their structural characteristics and synthetic methods. The building blocks include amine, aldehyde, borate esters, aryl cyanides, aromatic hydrocarbons, heterocyclic aromatics, etc. Additionally, the synthetic methods involve Friedel–Crafts alkylation chemistry, Sonogashira–Hagihara cross-coupling, phenazine ring fusion reaction, nitrile cyclotrimerization, Yamamoto coupling, boroxine anhydride formation reaction, borate ester formation reaction, etc. Benefiting from the well-defined structures and the controllable introduction of functional groups into the polymer skeleton, POPs are attracting significant attention from researchers in many different fields. Furthermore, the POP-derived carbon materials could not only inherit the aforementioned characteristics, but also exhibit good conductivity and superior stability. Consequently, POP-derived carbon materials always possess the following advantages: (1) efficient mass transfer properties, due to their porous structures; (2) higher electrocatalytic efficiency, attributed to their high surface area and highly exposed active sites; and (3) better intrinsic catalytic activity, resulting from their tunable electronic structures.

**Figure 1:**
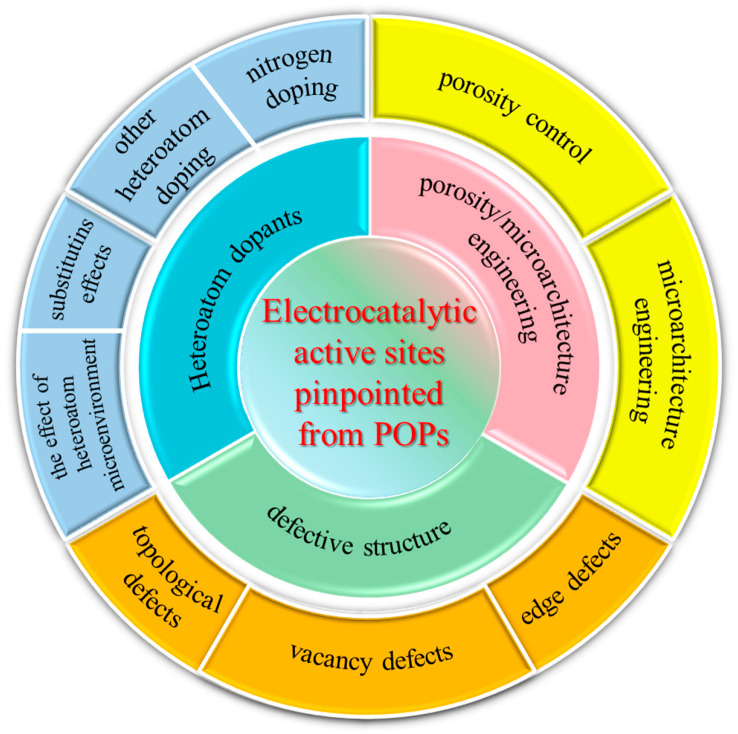
Overview of electrocatalytic active sites pinpointed from POPs.

In this review, we first explain the mechanism of the ORR and some basic electrochemical evaluation standards. Subsequently, we systematically revisit the evolution and progress of POP-derived carbonaceous ORR catalysts, emphasizing how this material system can serve as an ideal platform for recognizing, designing, and increasing the density of active sites at the molecular level for the electrocatalytic ORR. This core concept of our review serves to interconnect the material structure design and ORR performance evaluation, thereby offering meaningful insights to fellow researchers in the field. In this regard, we mainly focus on different types of active sites pinpointed from POPs, including heteroatoms, porosity/microarchitecture and defective structure (Figure 1). Finally, the future perspectives and challenges on designing new POPs for higher ORR performance are proposed. We believe this review will provide fundamental guidance for the development of POP-derived carbocatalysts for an electrocatalytic ORR and other related electrochemical reactions.

## 2. General Principles for ORR

### 2.1. Fundamental Mechanisms of Electrocatalytic ORR

The electrocatalytic ORR is a key electrode reaction for fuel cells, and also could singly act as a basic electrode in primary air-batteries to boost the conversion of chemical energy into electrical energy. In fact, the ORR process is quite complex and different intermediates—such as O*, OOH*, OH*, O_2_^2−^, O_2_^−^, and HO_2_^−^—will be produced during the reaction [8]. It is well known that the first step is the adsorption of oxygen molecule onto the catalyst surface, resulting the O_2_* intermediate (where * denotes the active site on the electrode surface). The next step decides the final product with two different ORR mechanisms [9]: (1) for the direct four−electron pathway, the O-O bond in the O_2_* intermediate breaks and an O* intermediate is formed. Then, the as-formed O* intermediate is reduced to OH* and to the final desorption of H_2_O; (2) for the series two-electron pathway, O_2_* is reduced successively into OOH* and then HOOH* (hydrogen peroxide on active site) before the O–O bond is cleaved, and the H_2_O_2_ becomes the main product. The difference between these two reaction paths can also be described as Equations (1)–(5):

In an acidic aqueous solution

For 4e^−^ ORR:

(1) $$O_{2}+4H^{+}+4e^{-}\;2H_{2}O\;\;\;\;\;\;\;\;\;\;E^{0}=1.23\;V\;(vs.\;RHE)$$

For 2e^−^ ORR:

(2) $$O_{2}+2H^{+}+2e^{-}\;H_{2}O_{2}\;\;\;\;\;\;\;\;\;\;E^{0}=0.70\;V\;(vs.\;RHE)$$

In an alkaline aqueous solution

For 4e^−^ ORR:

(3) $$O_{2}+2H_{2}O+4e^{-}\;2OH^{-}\;\;\;\;\;\;\;\;\;\;E^{0}=1.23\;V\;(vs.\;RHE)$$

For 2e^−^ ORR:

(4) $$O_{2}+2H_{2}O+2e^{-}\;H_{2}O_{2}+2OH^{-}\;\;\;\;\;\;E^{0}=0.76\;V\;(vs.\;RHE)$$

When the pH value is higher than 11.7, Equation (4) becomes the following equation [10]:

(5) $$O_{2}+H_{2}O+2e^{-}\;HO_{2}^{-}+OH^{-}\;\;\;\;\;\;\;\;\;\;E^{0}=0.76\;V\;(vs.\;RHE)$$

Generally speaking, the output voltage of a fuel cell, which undergoes the 4e^−^ pathway, is much higher than that of the 2e^−^ pathway. Consequently, the output power density and energy density, through the 4e^−^ pathway, will be dramatically increased, which is highly expected for the ORR in a fuel cell [11]. To meet this goal, a closer recognition and construction of the structural characteristics in an ORR electrocatalyst is urgently needed. According to the analysis of the reaction mechanism, the adsorption energy of the intermediates on the electrode surface should be moderate, since (1) when the adsorption of O* or OOH* is too strong, the desorption of H_2_O will be impeded, and the active sites on the electrode will be obstructed for the further adsorption of oxygen molecules; (2) when the adsorption is too weak, the electron/proton transfer step to the O_2_* intermediate will be difficult, and the generation of O* or OOH* will be blocked. Density functional theory (DFT) simulations show a volcano-shaped relationship between the ORR activity and the O*, OOH*, or OH* binding energy on the carbocatalysts. For example, Jiao et al. demonstrated that N- or B-doped graphene can bind to the ORR intermediates moderately, which showed superior activity than O-, P-, or S-doped graphene, or pristine graphene [12]. Essentially, it is the electronic structure that determines the binding energy between the intermediates and the electrode surface, which eventually will affect the electrocatalytic performance for the ORR. The detailed principle of the structural construction based on POP-derived carbocatalysts, as well as the structure–property relationships, will be discussed in the next section.

### 2.2. Electrochemical Evaluation for the ORR

Rotating disk electrodes (RDEs) and membrane electrode assemblies (MEAs) are the two common methods used to assess the activity and durability of an electrocatalyst [13]. Although the data obtained by membrane electrode assembly (MEA) measurements are more representative of fuel cells, the influence of operating conditions, as well as the assembly process of the catalyst layers, could not be fully unified in practice. In addition, MEAs could only discern the overall performance for the ORR, but the reaction mechanism could not be investigated [14]. As an alternative, with regard to RDEs and rotating ring-disk electrode (RRDE) measurements, the mass loading is only several tens mg cm^−2^ on the electrode holder, and the diffusion layer during the measurement is always between 5–50 µm, which guarantees the facile extraction of the kinetic parameters as well as the reaction mechanism for ORR. As a result, we will mainly focus on the RDEs and RRDE measurements in this review.

**Figure 2:**
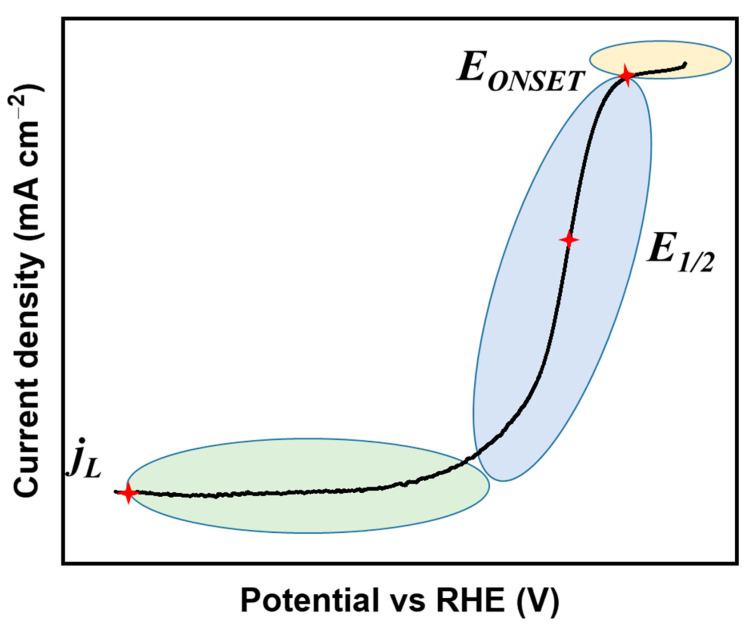
Typical polarization curve of oxygen reduction reaction for carbocatalysts.

For the electrode preparation, the carbocatalyst is typically dispersed into a mixture of deionized water, alcohol, and Nafion (5 wt%) by an optimized ratio. After an ultrasonic treatment, the obtained homogeneous ink is deposited uniformly onto the surface of a glass carbon RDE. A three-electrode system is always adopted for the testing procedure, with 0.1 M HClO_4_, 0.5 M H_2_SO_4_, or 0.1 M KOH as the solution. In an O_2_ -saturated solution, the cyclic voltammogram (CV) and polarization curve are measured by cyclic voltammetry and linear sweep voltammetry, respectively. Although the CV curve could be qualitatively related to the ORR activity by estimating the peak position of the cathodic segment, the result could not be used to compare different material systems because of the insufficient quantitative parameters. To alleviate the mass transfer loss during ORR testing, RDE is consistently rotated at 1600 rpm with the scan rate below 20 mV s^−1^. From the polarization curve (Figure 2), onset potential (E_onset_) and half-wave potential (E_1/2_) can be obtained directly, which are positively related to the catalytic activity. The kinetic current density (j_k_) of the catalysts without mass transfer effect (representative of the intrinsic activity) can be calculated by the Koutecky−Levich (K−L) Equation (6):

(6) $$\frac{1}{j}=\frac{1}{j_{k}}+\frac{1}{j_{L}}=\frac{1}{j_{k}}+\frac{1}{0.62nFAC_{0}^{*}D_{0}^{2/3}v^{-1/6}\varpi^{1/2}}$$

where j, j_k_, and j_L_ represent the measured current density (mA cm^−2^), kinetic current density (mA cm^−2^), and diffusion-limited current density (mA cm^−2^), respectively. In particular, the j_L_ is determined by the number of electrons transferred (n), the Faraday constant (F, 96 486.4 C mol^−1^), the concentration of dissolved O_2_ in solution (C_0_*, mol cm^−3^), the diffusion coefficient of O_2_ (D_0_, cm^2^ s^−1^), the kinetic viscosity of the solution (υ, cm^2^ s^−1^), and the rotation speed of the RDE (ϖ, rad s^−1^). The electron transfer number at the designated potential can be calculated from the slope of Equation (7), which provides valuable information for the ORR pathway:

(7) $$j_{L}=B\varpi^{1/2}=0.62nFC_{0}^{*}D_{0}^{2/3}v^{-1/6}\varpi^{1/2}$$

However, the K−L equation is not accurate when using porous carbocatalysts [15], due to the fact that the application scope for the K−L equation is mainly for the catalysts with flat surface. To assess the electron transfer number from the experimental data directly, RRDE is widely adopted to measure i_r_ (A) and i_d_ (A) at the ring and disk, respectively, according to Equation (8). Furthermore, the selectivity of H_2_O_2_ production could also be obtained according to Equation (9):

(8) $$n=\frac{4i_{d}}{i_{d}+i_{r}/N}$$

(9) $$H_{2}O_{2}\left(\%\right)=200\frac{Ni_{r}}{i_{d}N+i_{r}}$$

where N is the collection efficiency of the Pt ring.

## 3. Electrocatalytic Active Sites Pinpointed from POPs

Generally speaking, the structural design, to boost ORR performance for the carbocatalyst, is mainly categorized into three mainstream strategies: (1) doping heteroatoms into the carbonaceous skeleton to tune its electronic structure and eventually improve their catalytic activity; (2) engineering porosity and microarchitecture (i.e., the morphology control, hybridization, etc.) to facilitate the interaction with the ORR intermediates; (3) creating defects (i.e., topological defects, vacancy defects, and edge defects) to tune both the electronic structure and the adsorption/desorption of the intermediates [16]. Notably, the scenario for each structural design is diverse. For example, the doping elements comprise more than ten species, including B, N, O, S, Se, P, F, Cl, Br, I, etc. [17] Additionally, the defects may include pentagon, heptagon, zigzag edge, and armchair edge. In addition, these methods are not isolated, and a combination of two or more strategies could induce superior synergistic effects and even boost ORR performance to a level comparable to that of the commercialized Pt/C catalyst. Here, we summarize recent advances in how researchers have pinpointed the active sites in carbocatalysts, by constructing different types of POPs, and have achieved the desirable ORR performance.

### 3.1. Heteroatom Dopants

The introduction of heteroatoms into the pristine carbon materials could tune their electronic structures, charge distribution, and spin density, resulting in the modulation of their adsorption/desorption properties with ORR intermediates and electrocatalytic activity [18]. Particularly, the discrepancy between carbon and other heteroatoms in terms of their electronegativity, bonding states and atomic sizes, could create considerable active sites. It is well known that POPs can be easily and precisely synthesized to produce the desired heteroatoms, which are beneficial for the increase in catalytic site intensity and electron transfer on the carbonaceous framework [19], allowing POPs to act as efficient electrocatalysts for ORR. The recent progress in POP-derived carbocatalysts for the ORR are summarized, demonstrating their great potential in the recognition, construction, and intensification of active sites on the molecular level.

#### 3.1.1. Nitrogen Doping

In comparison to the carbon element, nitrogen possesses a similar atomic radius but with one more electron, and the typical N-doped graphene showed a variation in both the charge and spin density distribution of C and N atoms [20]. As a result, the adsorption energies of intermediates are changed on the active sites. Due to their tunable electronic properties and potential ORR electrocatalytic activities, N-doping has attracted significant attention and has been widely investigated.

**Figure 3:**
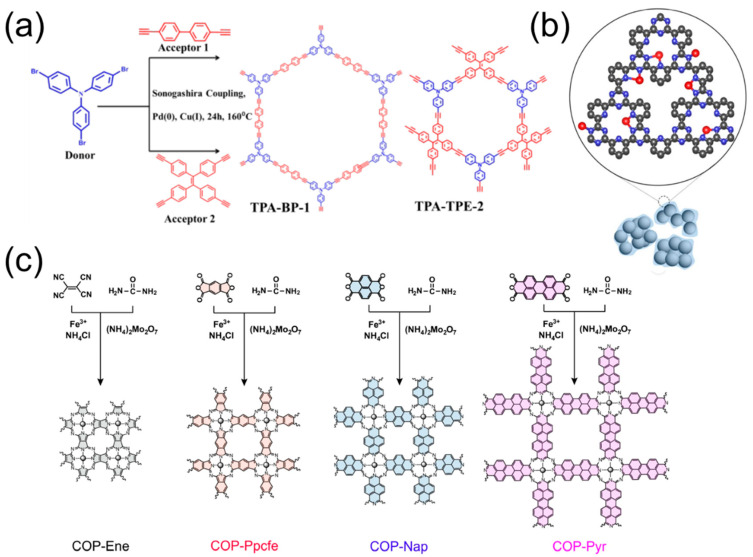
(**a**) Synthetic scheme towards fabrication of two redox active and semiconducting conjugated microporous polymers. Reproduced with permission [21]. Copyright 2018, Royal Society of Chemistry. (**b**) Schematic illustration of Pt-CTF/CP (blue—N, red—Pt, and black—C. Chlorine atoms are not shown for clarity). Reproduced with permission [25]. Copyright 2014, Springer Nature. (**c**) Formation of different kinds of Fe-N-C samples by tailoring monomers constructed with corresponding building blocks. Reproduced with permission [26]. Copyright 2022, Nature Publishing Group.

**Figure 4:**
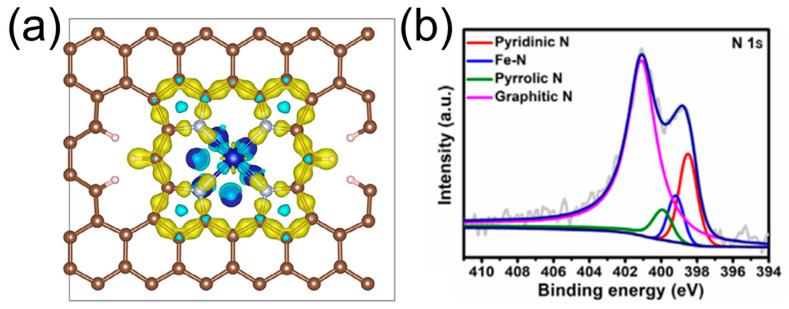
(**a**) Deformation charge density of Co_p_@CoNC, where the yellow areas and blue areas represent electron accumulation and electron loss, respectively. Reproduced with permission [27]. Copyright 2022, Elsevier. (**b**) XPS high-resolution N 1s spectra of the Fe-N-CNS sample. Reproduced with permission [28]. Copyright 2021, Elsevier.

The construction of nitrogen active sites on the molecular-level enables efficient adsorption of oxygen-active species. For example, the coupling of nitrogen-containing triphenylamine, and tetraphenylethene/biphenyl [21] (Figure 3a), produces TPA-BP−1- and TPA-TPE−2-conjugated microporous polymers (CMPs), which exhibit redox activity in the range of ORR. DFT calculations indicated that the triphenylamine core allowed for the homogeneous distribution of N active sites throughout the CMP skeleton, resulting in an increased amount of O_2_ adsorption on the catalyst surface. Moreover, POPs could serve as an ideal platform to anchor metal atoms as novel ORR catalysts, due to the following aspects: (1) the heteroatoms in POPs, especially the N heteroatoms with lone electron pairs, are inclined to coordinate with metal atoms; (2) the coordinating environment of metal atoms can be easily regulated in terms of their species, sizes, and spatial arrangement; (3) the obtained catalyst is extremely robust, due to the strong covalent bonding within the polymeric skeleton. As a result, the precise incorporation of foreign metal atoms into N-doped POPs has been widely investigated, which could produce the Fe-N_4_ and Co-N_4_ for superior ORR performance [22,23,24]. Kamiya et al. [25] explored the platinum-modified covalent triazine frameworks (CTFs) through the polymerization process (Figure 3b), where the confined Pt–N configuration in the Pt-CTF/CP (carbon particles) presented superior ORR activity and stronger methanol tolerance. Thus, the incorporation of Pt atoms into the CTF skeleton successfully addressed the long-standing methanol crossover effect for direct methanol fuel cells. Xiang et al. [26] synthesized a series of fully π-conjugated phthalocyanine-based covalent organic polymers (COPs) that could precisely load atomically dispersed Fe-N-C sites in a pyrolysis-free process (Figure 3c). Such a strategy avoids the unpredictable active-site configurations, making it an ideal platform for the study of the electronic configuration of FeN_4_ sites. Yang et al. [27] developed a Co_p_@CoNC catalyst composed of high-purity pyrrole-type CoN_4_ and cobalt particles (Figure 4a). Due to the regulated atomic and electronic structures, the high-purity CoN_4_ sites were demonstrated to be responsible for the improved ORR performance, including E_1/2_ as high as 0.84 V (vs. RHE) and a large peak power density of 188.8 mW cm^−2^ when assembled into a Zn-air battery.

The doping position in the carbonaceous skeleton could be precisely controlled by choosing suitable covalent organic polymers (COPs). As a pioneering work reported by Jiang et al. [28], the COP-derived carbocatalyst was synthesized by the Schiff base condensation between 1,3,5-triformylbenzene (TFB) and 1,4-diaminobenzene (PDA). During the subsequent one-step pyrosis process, abundant nitrogen content with a precise position, i.e., the regulated graphitic N and pyridine N, was successfully achieved (Figure 4b), demonstrating advanced ORR performance that was comparable to that of the commercial 20 wt% Pt/C catalyst. Nayak et al. [29] synthesized N-doped carbon materials from triazine-based porous organic polymers (POPQs) by using 2,6-diaminoanthraquinone and cyanuric chloride as the building blocks. The derivative N/POPQ800 carbocatalyst with enriched N content, which was distributed uniformly throughout the framework, exhibited a low E_onset_ = 0.832 Vvs. RHE. Furthermore, modulating the electron-donating/withdrawing ability, through the N-doping position, is imperative and worth investigating. Recently, Xu et al. [30] designed three kinds of dioxin-linked COPs with different electron-withdrawing groups (-CN/strong, -COOH/medium, and -CH_2_OH/weak). Through experimental measurement and theoretical calculation, the authors confirmed that the strong electron-withdrawing -CN, alongside the conjugated networks, could contribute to a more positive charge at the active sites and enhance the adsorption of intermediates, resulting in the facilitated adsorption of intermediates and promoted reaction kinetics for ORR.

Typically, N heteroatoms doped in carbon nanomaterials are classified into four different configurations: pyridinic, pyrrolic, graphitic, and pyridine-N-oxide species [31]. At the current point in time, the debate on the most efficient N species is ongoing. On the one hand, it is quite challenging to fabricate the N-doped carbocatalyst with only one type of the N species during the high-temperature treatment. On the other hand, the different carbon precursors always result in the carbocatalysts with unpredictable structures. Huang et al. [32] obtained ultrathin graphitized nitrogen-doped carbon nanosheets (NG-CNS) by pyrolysis of coordination polymers. The graphitization degree could be improved by the addition of FeCl_3_, which could help break the intermolecular force between the crystal structure during the pyrolysis treatment. More importantly, the configuration of N dopants could be adjusted during the subsequent ammonia treatment, resulting in a NG-CNS catalyst with excellent ORR performance (e.g., E_1/2_ = 0.80 V, a high diffusion-limited current density of 6.00 mA cm^−2^, and a small Tafel slope of 57.3 mV dec^−1^). However, the coexistence of pyridinic, pyrrolic, graphitic-N species has made it difficult to distinguish the exact N active center for ORR, regardless of the achievement of a better N-doped carbocatalyst. As a pioneering work, Haque et al. [33] systematically investigated and compared the ORR performance of pyridinic and graphitic N by a careful optimization of the pyrolysis temperature of the amino-functionalized metal–organic frameworks. Combining the experimental results and the gradient-correlated DFT calculations, they confirmed that the graphitic N sites exclusively catalyzed the ORR process through the 4e^−^ transfer pathway, while pyridinic and pyrrolic N were ineffective for the ORR. Afterwards, Jena et al. [34] chose 2,2′-dihydroxy-6′-isocyano-[1,1′-binaphthalene]-6-carbonitrile (BINOL-CN) as both a carbon and a nitrogen source, and synthesized four types of BINOL-CTFs with controlled N configurations. Combining the structural characterizations with the ORR performance, they demonstrated that the ORR activity depends exclusively on the number of quaternary-N species. The intricately designed BINOL-10-500 with the highest quaternary-N content, showing outstanding activity with the onset potential of 66 mV and an electron transfer number close to 4. By contrast, Zheng et al. [35] developed a facile solvothermal strategy for the fabrication of two kinds of covalent triazine polymers (CTPs); namely, the N-containing CTP (N-CTP) and O-containing CTP (O-CTP). Benefiting from the favorable thermal stability of CTPs, the large number of pyridinic-N sites in triazine rings can be sufficiently retained after pyrolysis. Therefore, the precisely designed CTP-derived N-doped carbon spheres (N/N-CS) exhibited outstanding activities for ORR, including a high E_1/2_ up to 0.865 V (vs. RHE), high 4e^−^ reduction selectivity, and excellent methanol tolerance. In addition, Cai et al. [36] found the incorporation of Cs atoms into the N-doped carbons could inject more electrons into the carbon skeleton and decrease the work function of the carbocatalyst. Consequently, the adsorption of the intermediates, e.g., OOH*, is facilitated and the ORR activity is improved.

#### 3.1.2. Other Heteroatom Doping

**Figure 5:**
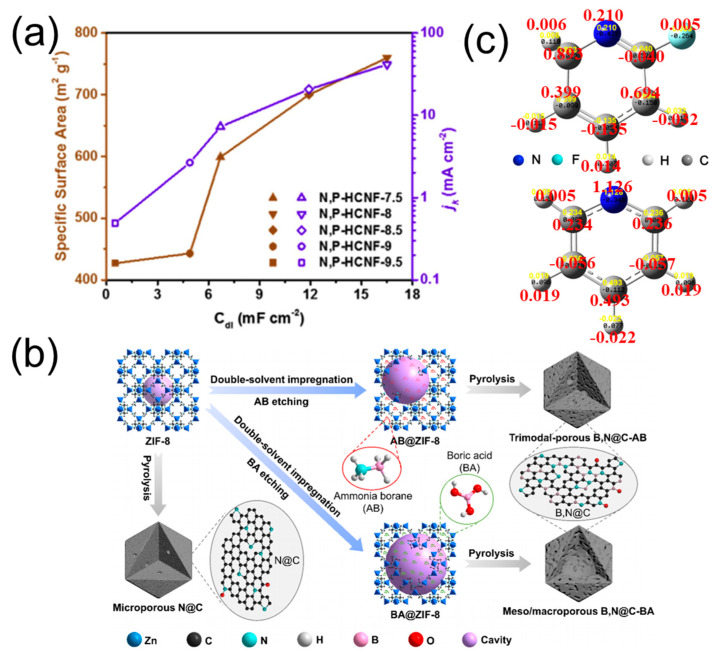
(**a**) The relationship between BET specific surface areas, C_dl_ and j_k_ of N, P−HCNFs. Reproduced with permission [38]. Copyright 2019, Elsevier. (**b**) Schematic diagram of the fabrication of B, N@C nanocages. Reproduced with permission [43]. Copyright 2022, Springer Nature. (**c**) Fluorine-induced redistribution of the charge and spin [45]. Copyright 2022, Elsevier.

When nitrogen-doped carbon materials are co-doped with phosphorus, the valence orbital energy levels of the adjacent carbon atoms are impacted, resulting in an enhanced oxygen reduction reaction (ORR) reactivity through the synergistic effects of phosphorus and nitrogen heteroatoms [5]. To synthesize a nitrogen/phosphorus co-doped carbocatalyst, a Schiff base reaction was performed between 1,3,5-tri-(4-aminophenyl) benzene (TAPB) and dimethoxy-terephthaldehyde (DMTA), followed by carbonization and phosphorization [37]. The resulting “C^+^” centers are the dominant active sites, due to the strong synergistic effects of the N–C, P–C, and P–N chemical bonds. Nitrogen doping into graphitic carbon resulted in increased electron density towards the nitrogen-doped region, facilitating electronic and ionic conductivity. Phosphorus doping not only increased surface defects for enhanced ORR performance, but also stabilized the C-N species and improved cycling stability. Furthermore, Gao et al. [38] demonstrated that the incorporation of P elements into N-doped carbon materials could enlarge a specific surface area (Figure 5a) to improve intrinsic catalytic activity and reduce the ionic resistance in pores, leading to the superior mass transfer property for ORR. It is worth noting that P sources can also be covalently introduced into the carbon skeleton using P_2_O_5_ as a carbonizing agent [39], which can facilitate a series of structural evolutions, including 2D morphology molding and a hierarchical pore-forming pathway.

The difference in electronegativity between boron (B) and carbon (C) is noteworthy, with B possessing a lower value (2.04) than C (2.55). Consequently, B can substitute C in the carbon lattice and modify the charge density within the carbocatalyst. The incorporation of B/N co-doped carbon materials has been shown to have great potential for energy storage and conversion devices, as evidenced by previous studies [40,41]. Kahan et al. [42] synthesized a range of B/N-doped polycyclic aromatic hydrocarbons (PAHs) through the bottom-up approach. It was observed that the doubly B-doped and B/N co-doped PAHs exhibited the most active oxygen reduction reaction (ORR) property, due to their lower LUMO energies. To ensure the uniform distribution of B within the carbon framework and to enhance the efficiency of mass transfer during the electrochemical reaction, Wang et al. [43] developed a host–guest chemical strategy that involved the incorporation of B sites into the cavities of metallic porous organic polymers (Figure 5b). This approach resulted in a high B doping content of 15%. The hierarchical porous structure and interaction between B/N heteroatoms has synergistically contributed to the remarkable ORR performance of the resulting B, N@C carbocatalyst, which exhibited an E_1/2_ as high as 895 mV. Typically, B and N are randomly distributed in a B/N co-doped carbon skeleton, resulting in a disordered structure that limits a deeper understanding of the relationship between their chemical structure and properties. Ishii et al. [44] investigated the selective generation of B-N-C moieties, with well-defined chemical structures and controllable amounts, by exploiting the dehydration reaction between 1,8-diaminonaphthalene (dan) and boric acid. The experimental results revealed that the ORR activity of the material is determined by the surficial B-N-C moieties.

Nitrogen (N) doping has been widely studied for its capability to enhance the adsorption and dissociation of oxygen molecules, thereby improving the oxygen reduction reaction (ORR) performance. However, the protonation of pyridinic-N species in acidic media can lead to inferior ORR performance. Fluorine (F) doping, on the other hand, has shown promising results for improving the acidic ORR performance of carbocatalysts, due to its high electronegativity (χ = 3.98), which induces polarization and charge delocalization on neighboring carbon atoms. DFT calculations by Zhang et al. [45] revealed that incorporating F into the pyridinic-N structure significantly improves charge and spin densities to 0.521 and 0.694, respectively (Figure 5c). This theoretical result is consistent with the experimental acid ORR performance trend of N/F co-doped carbocatalysts. Sun et al. [46] synthesized a N/F-containing COP through Schiff base reaction between 1,3,5-tri-(4-amino-phenyl) benzene (TAPB) and 2,3,5,6-tetrafluoroterephthaldehyde (TFTA). Through comprehensive investigations, they demonstrated that the dual doping of graphitic N and covalent F exhibited high electrocatalytic activity and an extremely low free energy barrier over a wide range of potentials (vs. RHE), due to the reticular chemistry and supramolecular interactions that maintain the polymeric skeletons of the COPs.

**Figure 6:**
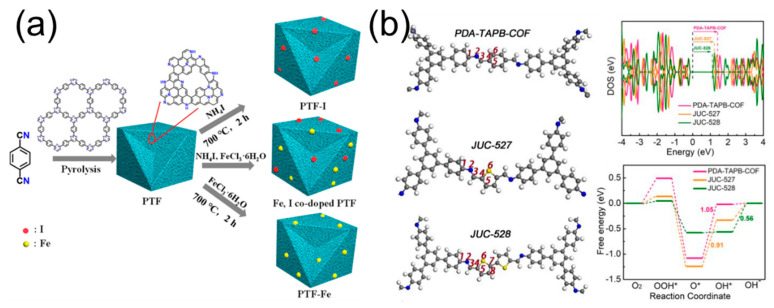
(**a**) Synthesis of N/I co-doped catalyst. Reproduced with permission [47]. Copyright 2019, American Chemical Society; (**b**) structural models and the related density of states and free energy diagrams of PDA-TAPB-COP, JUC-527, and JUC-528 samples. Reproduced with permission [49]. Copyright 2020, American Chemical Society.

The doping of both iodine and sulfur elements, in carbon-based oxygen reduction reaction electrocatalysts, plays a crucial role in enhancing their performance. Iodine is widely recognized as an important dopant in enhancing the electrical conductivity of carbon nanomaterials by increasing the hole mobility of the carbon skeleton [47]. Su et al. [48] have reported a N/I co-doped catalyst, prepared through an I_2_-assisted pyrolysis strategy from zeolitic imidazolate framework-8 (ZIF-8), which consists of Zn^2+^ and 2-methylimidazole (Figure 6a). The introduction of gas-phase I_2_ increased both the electrochemical surface area and meso/macropores, resulting in enhanced oxygen reduction reaction (ORR) mass transfer and excellent ORR activity, as evidenced by a high E_1/2_ of 0.868 V (vs. RHE), j_L_ of 5.84 mA cm^−2^, and good stability with 96% current retention after 15,000 s. Based on the precise construction of two metal-free thiophene-S COPs (MFTS-COPs, JUC-527 and JUC-528), Li et al. [49] verified that the pentacyclic thiophene-S building blocks were the efficient active centers for the ORR (Figure 6b). In particular, the JUC-528 with bi-thiophene-S structures shows better ORR activity (E_1/2_ = 0.70 V vs. RHE) than one thiophene-S-constructed JUC-527 (E_1/2_ = 0.63 Vvs. RHE), indicating the ORR performance is proportional to the number of thiophene-S structures.

In recent years, various forms of multiple-heteroatom-doped carbocatalysts, particularly tri-doped carbocatalysts, have been reported to exhibit superior oxygen reduction reaction (ORR) performance through the synergistic effects of their unique electronic structures. The underlying principle of structural design aims to combine the advantages of the different heteroatoms to enhance ORR activity. For instance, multi-heteroatom-doped N, S and P graphene (NSP-Gra) were shown to function as an excellent ORR/OER electrocatalyst, due to the presence of active sites composed of the multi-heteroatom dopants [50]. Zheng et al. [51] synthesized N-, P-, and F-self-doped carbon spheres by polymerizing a N/P-containing HPPC monomer and a F-containing TFHQ monomer to produce a tri-doped CTF, which was subsequently pyrolyzed. The heteroatoms were found to be uniformly distributed throughout the carbon skeleton. Due to the synergistic modulation of the electronic structure of the carbocatalyst, the N/P/F-tri-doped model exhibited superior ORR performance across a wide range of pH conditions, including alkaline aqueous (0.1 M KOH), acid aqueous (0.5 M H_2_SO_4_ and 0.1 M HClO_4_), and neutral aqueous (0.1 M PBS).

In fact, the heteroatoms present in carbocatalysts, derived from porous organic polymers (POPs), not only function as direct active sites for enhancing oxygen reduction reaction (ORR) performance, but can also serve as anchoring sites for other active centers, such as single metal atoms. For instance, a N-doped holey carbon material, coordinated with Fe atoms (Fe-NHC), displayed a significantly higher ORR half-wave potential of 0.89 V (RHE), compared to Co- or Ni-coordinated carbocatalysts, highlighting the efficacy of heteroatoms in promoting an ORR [52]. Wei et al. [53] introduced a new approach for the synthesis of N/P co-doped mesoporous carbon nanospheres, using covalently cross-linked poly(hexachlorocyclotriphosphazene-tannic acid-BPS) (where BPS stands for 4,4′-sulfonyldiphenol) nanospheres (PSTA) as a precursor material. The resultant nanospheres were subsequently chelated with metal ions to produce uniformly dispersed single atom Co-N_2_P_2_ sites, which demonstrated enhanced electrocatalytic activity towards the oxygen reduction reaction (ORR).

#### 3.1.3. Substitutions Effects

Although the electronic environment of POPs is easily modulated by incorporating heteroatoms into the molecular building blocks, the resultant covalent skeletons are always accompanied by a complex polymeric configuration, i.e., the heteroatom contents, spatial extensibility, and bonding angle. More importantly, the ORR performance is susceptible to these specific structure-induced effects, such as the heteroatom number and location, steric hindrance, and bonding angle. As a result, it is imperative to design a heteroatom-free regulation strategy to accurately identify the substitution effects. Building upon this concept, You et al. developed carbon-based COPs incorporating heteroatom-free methyl groups (MGs), composed exclusively of carbon and hydrogen atoms, into the polymer skeleton. Through a combination of in situ Raman spectroscopy and DFT calculations, the authors observed that the MG-bound skeleton could induce ortho activation, leading to the identification of the ortho carbon (site-5) adjacent to the MGs as the active centers for the oxygen-reduction reaction [54].

Singh et al. synthesized two donor–acceptor-based CMPs (TAPA-OPE-mix and TAPA-OPE-gly) via a Schiff base condensation reaction [55]. They observed that the asymmetric and symmetric bola-amphiphilic nature of oligo-(p-phenyleneethynylenes) (OPE) struts has resulted in not only the distinct nano-structuring and morphologies in the products, but also the different electrochemical behavior and ORR performance. Impressively, more hydrophilic interior pores in TAPA-OPE-gly samples could facilitate substrate transfer towards the catalytic center, and a higher current density of 4.8 mA cm^−2^ was achieved at the rotation speed of 2500 rpm, compared with that of 3.9 mA cm^−2^ for TAPA-OPE-mix.

Recently, Wu et al. [56] demonstrated that the radical substitutions incorporated into a 2D COP could lower the energy gap to 0.88 eV, and reduce the LUMO energy level to −4.72 eV, resulting in high electro-catalytic activity (E_1/2_ = −0.27 V vs. Ag/AgCl) and durability (only a slight current density loss of 12% after 40,000 s) toward ORR. Such novel π-conjugated COP, based on the stable polychlorotriphenylmethyl (PTM) radical, was synthesized via liquid/liquid interfacial homo-coupling, and the subsequent deprotonation and oxidation of the polychlorotriphenylmethane unit produced the PTM radical.

For metal−nitrogen/carbon ORR catalysts (M–N/C), the proton-coupled electron transfer (PCET) reaction, especially the first elemental step, is the rate-determining step (RDS) [57,58]. To facilitate the ORR process, an electrocatalyst with H^+^ tuning capability is of critical scientific significance. Liu et al. [59] decorated polyaniline (PANI) onto a Fe-N/C catalyst and thus, successfully constructed a protophilic surface. In comparison with the traditional Fe-N/C catalyst, the PANI-decorated one was demonstrated to be capable of enriching the interfacial H^+^ along the protophilic surface, and a 20 mV positive shift in half-wave potential was achieved with a tripled TOF (from 0.46 to 1.28 e s^−1^ sites^−1^). Likewise, the decoration of metal-containing fragments was also investigated by Peles-Strahl et al. [60], where the polytriethynyl-benzene (PTEB) aerogel was polymerized by using 1,3,5-triethynylbenzene (TEB) as the building block. Interestingly, imbedding the copper-containing bipyridine moieties into the aerogel activated its ORR catalytic property, whereas the one without bipyridine presented negligible ORR activity.

#### 3.1.4. The Effect of Heteroatom Microenvironment

Despite significant efforts towards the incorporation and modulation of heteroatoms in carbocatalysts for the oxygen reduction reaction (ORR), it has been increasingly recognized that the microenvironment surrounding heteroatoms also plays a crucial role in enhancing ORR performance. In fact, carbon materials with different topological structures, hybridized states, and spatial morphologies can exhibit considerable variations in ORR performance, even with comparable nitrogen content and configurations. Our group designed a series of nitrogen-enriched carbon composites (NEC), with controllable nitrogen configuration and regularly varied sp^2^ carbon content, through a rationally designed Schiff base chemistry approach [61]. Combined with the DFT results, we found that when the quaternary nitrogen content is identical, the sp^2^ carbon content had a great impact on the vertical ionization energies (VIE), which is proportional to the ORR performance. Furthermore, the electron-conductive quaternary-N site (ENS) is proposed as an important structural parameter, which integrates the contents of quaternary nitrogen with sp^2^ carbon. The ENS is defined as quaternary N sites with a fully electron-transporting region, and its calculation formula is:

ENS = [Cont (sp^2^ carbon)]^2^ × Cont (quaternary nitrogen)

**Figure 7:**
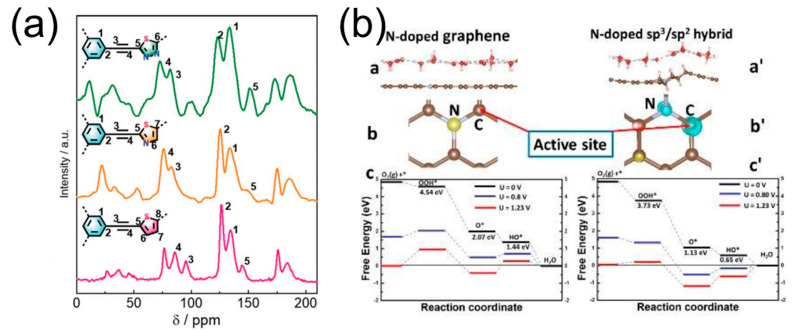
(**a**) The solid-state ^13^C NMR spectrum of CMP-Tp, CMP-Tz, and CMP-Tdz. Reproduced with permission [62]. Copyright 2023, Wiley-VCH (**b**) Comparison of Gibbs free energy for N-doped graphene and the N-doped sp^3^/sp^2^ hybrid. Reproduced with permission [63]. Copyright 2019, Wiley-VCH.

In this formula, [Cont] represents the content of the specific structure parameters, i.e., sp^2^ carbon and quaternary nitrogen. It is important to note that it is not the atomic site itself that possesses conductivity, but rather the surrounding sp^2^ carbon environment that contributes to the overall conductive nature of the electro-conductive quaternary-N site. Particularly, ENS showed a well-fitted result with the value of VIE, and it agrees well with the experimental ORR performance, which could be adopted as an important structural parameter to predict the electrochemical property of N-doped carbocatalysts. Afterwards, Zhao et al. investigated the effect of sp-hybridized C atoms as the main microenvironment in the S- and N-co-doped CMPs for the ORR [62]. The S-containing thiophene heterocycle (CMP-Tp) and S- and N-containing thiazole/thiadiazole heterocycles (CMP-Tz, CMP-Tdz) were fabricated through a facile one-step Sonogashira coupling reaction with a controllable alkynyl group (Figure 7a). Interestingly, the DFT calculations demonstrated that the high catalytic performance of these catalysts resulted from the sp-hybridized C atom, which is activated by their adjacent heteroatom structures.

The interface of sp^3^/sp^2^ carbon could play a critical role in the practical performance of the ORR carbocatalyst. Gao et al. [63] adopted the direct pyrolysis method of ionic liquid polymers to construct the controllable sp^3^/sp^2^ interface (Figure 7b). The DFT calculations indicate that the N dopant interacted with the sp^3^/sp^2^ interface and optimized the Gibbs free energies of intermediate species adsorption process. Moreover, the experimental results matched well with the theoretical cases that the 3D carbocatalyst, with abundant N dopants and a modulated sp^3^/sp^2^ carbon hybrid, enhanced the overall ORR performance.

**Figure 8:**
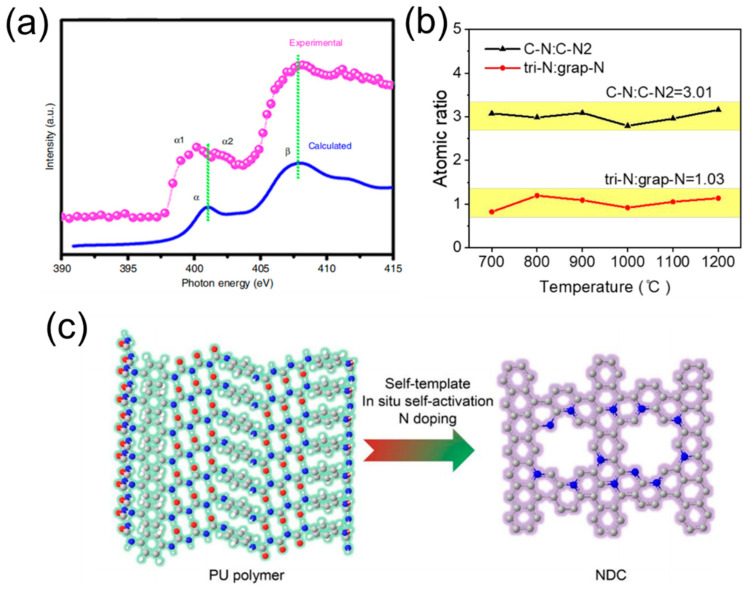
(**a**) The calculated curves of N K-edge X-ray absorption near-edge structure (XANES) spectra for N-HsGDY−900 °C. Reproduced with permission [66]. Copyright 2018, Springer Nature. (**b**) Atomic ratios of C-N:C-N2 and tri-N:grap-N. Reproduced with permission [67]. Copyright 2020, American Chemical Society. (**c**) Molecular structure illustration from PU to NDC. Reproduced with permission [69]. Copyright 2020, Wiley-VCH.

Graphdiyne (GDY) is a kind of carbon allotrope, consisting of both sp^2^ and sp hybridized carbon atoms. Previous investigations have demonstrated that the acetylenic linkages in the molecular skeleton create C^+^ in GDY, which could serve as active sites for ORR [64,65]. Lv et al. [66] synthesized hydrogen-substituted graphdiyne (HsGDY) through a cross-coupling reaction, starting from triethynylbenzene as the building block (Figure 8a). DFT results indicated that the pyridinic N-HsGDY possessed a much smaller free energy change (0.89 eV) for the rate-determining step, compared with that of the pyridinic N-graphene (1.17 eV), implying that the former is more active for ORR than the latter. Considering the different chemical environments and the similar N species in graphdiyne and graphene, it can be deduced that the discrepancy of the ORR performance may originate from the sp hybridized carbon atoms.

Apart from the hybridized state of the carbon atoms, the topological structure also exhibits a significant impact on the N heteroatoms to alter the ORR activity. Ju et al. [67] constructed several theoretical models with different coordination environments (Figure 8b), and found that the C-N_2_ site exhibited a more positive charge and a stronger oxygen bonding strength than the C-N site, which implied the C-N2 possessed a superior ORR activity compared with C-N sites. Accordingly, they synthesized a series of CTFs through the typical ionothermal method, in which the unique triazine ring structure (C_3_N_3_) can be regarded as a superstructure consisting of three C-N_2_ sites. Compared with the C-N sites in the N-doped graphene catalyst, the C-N_2_ sites were observed to exhibit a higher ORR activity. This provides evidence that the short-range, ordered topological structure may regulate the electronic structure of a carbocatalyst more efficiently than the randomly introduced N dopant.

Other than the hybridization state of carbon element, the crystalline structure could also affect the function of N active sites for ORR. Generally, the crystalline structure of carbon materials is classified into amorphous, graphitic, and turbostratic states [68]. Specifically, the turbostratic state represents an intermediate phase that lies between the amorphous and graphitic states, exhibiting a not short-range, ordered graphitic stacking but a long-range, disordered structure. When incorporated into such atypical carbon nanostructures, N dopants are expected to be effectively utilized, resulting in enhanced ORR performance. Inspired by a block copolymer (BCP) strategy for the fabrication of functional nanocarbon materials, Lai et al. [69] synthesized precursor-controlled turbostratic carbon nanomesh with abundant N active sites (Figure 8c). The authors employed DFT calculations to investigate the chemical environment of the N active site. Specifically, they found that the carbon edge defect from the turbostratic carbon skeleton, integrated with the graphitic valley N, could significantly lower the ORR energy barrier to 0.56 eV, resulting in a high kinetic current density of 9.46 mA cm^−2^ and a half-wave potential of 0.860 V (vs. RHE) in practical applications. Of great importanceid the fact that this molecularly designed carbocatalyst provides critical insights for tailoring the turbostratic structures of N-doped carbon materials to gain a deeper understanding of the ORR electrocatalytic mechanism.

### 3.2. Microarchitecture Engineering

Despite the remarkable advances in ORR performance facilitated by heteroatom-doped carbocatalysts, developing novel carbon materials for efficient electrocatalysis remains a daunting task. In most cases, researchers mainly focus on the design of precursors and the doping methods, yet the heteroatom-doped carbocatalysts with rationally engineered microarchitecture is always ignored, which could also have an important influence on the electrocatalytic performance. Generally speaking, these microarchitectures include the porosity modulation, morphology control and dimension adjustment.

#### 3.2.1. Porosity Modulation

The hierarchical porous structure, with its facile inner pore transferability, large accessible surface areas, short diffusion paths, and low density, is a desirable feature for catalytic reactions [70,71]. In the electrocatalytic ORR process, the porous structure and void space play a critical role in facilitating charge and ion transfer within the bulk carbon skeleton [72]. Therefore, the development of carbon materials, derived from POPs with hierarchical porous structures, holds tremendous potential for the creation of advanced ORR electrocatalysts. For example, by assembling a covalent triazine framework (CTF) network on the surface of melamine-cyanuric acid (MCA) supramolecular crystal [73], N-doped hollow carbon nanoflowers (N-HCNFs) with abundant porosity were developed as a metal-free electrocatalyst. Compared to traditional hard/soft templates such as SiO_2_ and polystyrene (PS) spheres, the use of MCA as a template offered unique advantages, including its rich surface chemistry and ease of removal. As a result, a hierarchical porous structure, consisting of micro- and mesopores, was formed during the volatilization of the MCA crystals, resulting in the creation of abundant surficial N active centers. The N-HCNFs synthesized using this method demonstrated exceptional electrocatalytic activity, achieving a high current density of 212 mA cm^−2^, at a peak power density of 136 mW cm^−2^, when used as the cathode for Zn-air batteries.

Choosing suitable building blocks could produce isometric CTFs with desirable pore geometry. For instance, two CTFs with different porous structures were prepared by the bicyano-based isomeric building blocks [74], and the subsequent carbonization afforded N-doped carbocatalyst with adjustable micro/meso pores. When used as a cathode for Zn-air batteries, the carbocatalyst exhibited a high-power density (125 mW cm^−2^) and specific capacity (651 mAh g^−1^). In a recent study by Li et al. [75], a hierarchical porous carbocatalyst was synthesized using a dual pore-forming approach with a focus on the pore-forming reactant. Specifically, nanoscale melamine resin spheres were employed to generate mesopores, while, in situ, reduced metallic zinc was utilized for micropore formation. The resulting micro/mesoporous structure enables superior mass transfer during the ORR process, resulting in a high power density of 176 mW cm^−2^, when implemented as the cathode for Zn-air batteries.

In general, the porous structure and graphitic skeleton are considered to be two crucial factors influencing ORR properties. The graphitic skeleton enables fast electron transfer and, therefore, improved reaction kinetics during electrochemical reactions. However, the pursuit of both high surface area and a highly graphitized skeleton is constrained by a trade-off relationship between these two structures. This is because the opposite synthetic conditions are required to produce these two structures—high graphitization is easily achieved at elevated temperatures, while porous structures are inclined to collapse under such conditions. To address this challenge, Chen et al. [76] employed amine aromatic organoalkoxysilane as an all-in-one precursor and FeCl_3_·6H_2_O as an active salt template, where the FeCl_3_·6H_2_O could facilitate the crystalline structure while the cross-linking reaction of alkoxysilane group enabled the highly porous structure. As a result, when used as the cathode for the Zn-air battery, the optimized GPCNSs carbocatalyst exhibited much smaller voltage drops between 1 to 20 mA cm^−2^, compared with that of the Pt/C catalyst, confirming accelerated reaction kinetics.

#### 3.2.2. Morphology Control

**Figure 9:**
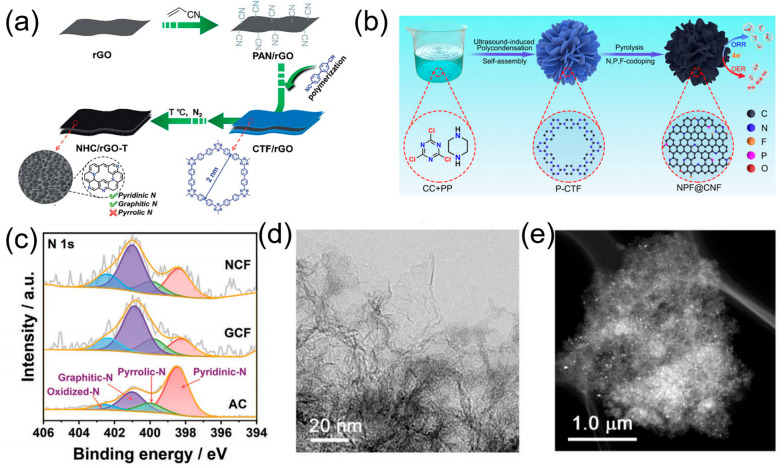
(**a**) Illustration of rational stepwise fabrication of the sandwich-like layered composite. Reproduced with permission [77]. Copyright 2017, Royal Society of Chemistry. (**b**) Procedures of Ultrasound-triggered assembly of P-CTF for synthesizing the tri-doped carbon nanoflowers. Reproduced with permission [78]. Copyright 2021, American Chemical Society. (**c**) High-resolution N 1s XPS spectra for NCF and the reference sample. Reproduced with permission [79]. Copyright 2021, Wiley-VCH (**d**) TEM and (**e**) HAADF-STEM of Fe-SCNS. Reproduced with permission [80]. Copyright 2020, Wiley-VCH.

The sandwich-like carbon materials exhibit nanometer thickness, infinite length, and superb charge transport pathways. Notably, the carbon framework, which constitutes the entirety of the bulk material, is substantially exposed to the electrolyte and can participate in the catalytic ORR. However, the intricate synthesis process of the carbocatalysts, derived from POPs, limits their large-scale applicability. To overcome this challenge, Jiao et al. [77] reported a novel carbon nanosheet using 4,4′-dicyanobiphenyl (DCBP) as a building block, via a trimerization reaction. The researchers employed a subtly designed template of polyacrylonitrile-decorated reduced-graphene oxide (PAN/rGO) to create sandwich-type composites consisting of covalently linked DCBP-based CTF, grown on both sides of the PAN/rGO (Figure 9a). The resulting 2D sandwich morphology, combined with the high surface area, hierarchical pores, and active nitrogen species, gave exceptional ORR activity (E_1/2_ = 0.83 V vs. RHE) and ultrahigh stability (97% current density retention after 20,000 s) on the layered N-carbocatalyst, along with excellent methanol tolerance.

Owing to their superior structural stability and high carbonization yields, 3D polymer nanoflowers have been widely recognized as highly desirable precursors for the fabrication of advanced carbocatalysts. Accordingly, Zheng et al. [78] reported piperazine-containing P-CTF as the nanosheets, which were converted into 3D N-, P-, F-tri-doped carbon nanoflowers (Figure 9b). It is worth mentioning that the ultrasound-triggered polycondensation and assembly played an important role in the conversion of morphology from the 2D nanosheets into the 3D flower-like structure. The obtained carbocatalyst showed an impressive half-wave potential of 0.85 V (vs. RHE) and remarkable stability, with over 90% current retention even after 50,000 s.

From a morphology engineering perspective, carbon nanosheets/flakes exhibit competence in providing active edge defects and exposing heteroatom centers. However, the amount of edge structure, when compared with the basal planes, is still restricted. Creating out-plane edges, based on the large-area basal plane, is attracting researchers’ attention. Zhang et al. [79] reported novel edge-suffused N-doped carbon nanoflakes (NCF), using sandwich-like ZIF-8 (an archetypical Zn-based metal-organic framework)/MnO_2_ as precursor (Figure 9c). The assembled aqueous Zn-air battery based on the optimized NCF exhibited an ultrahigh peak power density of 173 mW cm^−2^, which could be resulted from the synergism of pyridinic-N/graphitic-N dipole on the additional out-plane edges.

Three-dimensional superstructures with specific functionalities are also attractive, considering their vast potential in energy storage and conversion fields. Compared with the 2D nanosheets, which may possess inferior conductivity and mass transfer properties, a 3D superstructure could be a rational choice to optimize electrochemical performance, by decorating various functional species and developing sufficient contact interface. Zou et al. [80] synthesized a centimeter-scale 3D superstructure, composed of carbon nanosheets (SCNSs), by pyrolysis of a MOF nanostructure, which exhibited a honeycomb-like morphology (Figure 9d,e). After immobilizing metal nanoparticles, the Fe-SCNSs electrode showed an extremely high E_1/2_ of 0.89 V (vs. RHE) and a large limiting current density of 6.08 mA cm^−2^ for an ORR in alkaline electrolyte. Carbon nanocages with active sites have recently garnered significant attention, and introducing opening pores on the walls of the nanocages could promote mass transfer and enhance overall electronic conductivity. Hou et al. [81] created a hydrangea-like superstructure of open carbon cages through a self-templated strategy, in which core–shell MOF-comprised Zn-MOF and Co-MOF were chosen as the building blocks. After a direct morphology-controlled thermal treatment, a hydrangea-like nanocage, interconnected by carbon nanotubes was developed. The smoothed mass transfer walls allowed a high peak power density of 190.3 mW cm^−2^ when utilized as a cathode in a Zn-air battery. Wang et al. [82] developed dual-shelled carbon nanocages, doped with Co, N and S, by wrapping ZIF-67 with trithiocyanuric acid (TCA), in which the thickness of the shell can be controlled by modulating the interaction between the building blocks. As a result, the optimized Co-N/S−DSHCN-3.5 nanocage exhibited outstanding ORR activity with high E_1/2_ observed in both alkaline and acid electrolytes. In addition, Iglesias et al. [83] reported a metal-free electrocatalyst consisting of graphitized N-doped single-wall carbon nanohorns (CNHs), using the dopamine hydrochloride as the building block. The microporous volume in the CNHs textures enhanced the penetration of oxygen molecules across the active sites, while hindering the further reduction of the intermediate H_2_O_2_, which favored a 2e^−^ ORR path.

#### 3.2.3. Dimension Adjustment

COPs are featured by their molecular designability and abundance of functional sites. Generally speaking, most of the COPs possess 2D topology, with the catalytic sites located on the basal planes; however, the strong π-π stacking between the COP layers may impede the efficient utilization of the active sites. To maximize the utilization of the buried catalytic sites, exfoliating the 2D COPs into few-layer, or even single nanosheets, represents an effective strategy. [84]. For example, Royuela et al. [85] reported a novel naphthalene diimide-based COP (NDI−COP), which was successfully exfoliated into COP nanosheets (CONs), and demonstrated great potential for ORR in alkaline media. The incorporation of NDI functioned as an electron-poor building block, enabling the energetically favorable catalyst to accept electrons from the oxygen molecule. However, due to the less exposure of active sites, the ORR performance is inferior, including a reduction peak at −0.38 V (vs. SCE), an onset potential at −0.25 V (vs. SCE), and an electron transfer number of 3.6. This outcome suggests that the efficient utilization of the active center can significantly impact the catalytic activity for ORR, and the inadequate exfoliation of 2D nanosheets may still hinder the enhancement of ORR performance.

Furthermore, ORR catalysts based on COPs are typically characterized by inferior electronic conductivity, and subsequent heat treatment is often required to improve the charge transfer efficiency and enhance ORR activity. However, direct pyrolysis of COPs inevitably results in the formation of 3D carbon entanglements, leading to the loss of their 2D structural characteristics and their potential advantages for the ORR process. To hold the 2D structure while increasing the conductivity, Xu et al. [86] developed a 2D COP with a suitable phytic acid (PA) template to guide the pyrolysis, which is characterized by high conductivity, hierarchical porosity, and abundant N- and P-doped catalytic centers. Remarkably, the authors confirmed that the PA template played a critical role in converting the COPs into 2D carbon sheets, creating the nanosized 2D layers and avoiding the structural aggregate or even collapse. The COP-derived carbocatalyst showed excellent performance with exceptional onset potential (0 V vs. Ag/AgCl), half-wave potentials (−0.11 V vs. Ag/AgCl), high-limit current density (7.2 mA cm^−2^), low Tafel slope (110 mV decade^−1^), long-time stability (95 % current density retention after 5.5 h) and methanol tolerance.

By contrast, 1D COPs not only inherit the abundant active sites similar to the 2D COPs, but also exhibit additional catalytic centers for ORR, due to the presence of more edge sites. To fabricate 1D COPs, two methods are always implemented: (1) Synthesizing the COPs alongside a one-dimensional template. For example, Liu et al. [87] developed coaxial one-dimensional van der Waals heterostructures (1D vdWHs) using the carbon nanotube as core and a thickness tunable thienothiophene-pyrene COP as a shell. The 1D structure reduced the charge transfer barrier between the active sites and the adsorbed oxygen intermediates, which dramatically improved the catalytic ORR activity. Li et al. [88] adopted a thermal polymerization method to coat a COP (based on the building blocks of hexachlorocyclotriphosphazene and dicyanamide) onto the CNT. The obtained 1D N/P-co-doped nanohybrids, with highly porous structure and abundant graphitic-N and −P sites, presented a high E_1/2_ up to −0.162 V (vs. RHE), high current density of 6.1 mA cm^−2^, and good stability (83% retention after 36,000 s) for an ORR in alkaline solution. (2) Inducing the reaction based on non-linear edges and suitable high-symmetry vertices [89]. Encouraged by this structural concept, Hu’s group recently synthesized two 1D COPs, based on 4,4′-(2,2-diphenylethene-1,1-diyl) dibenzaldehyde (TPEDH) linkers [90]. Restricted by the molecular symmetry, the dibenzaldehyde in the TPEDH guaranteed that the TPEDH-based COP only extend along the 1D direction. Consequently, the 1D COPs were confirmed to be capable of catalyzing the ORR with excellent activity, without requiring the introduction of additional building units containing heteroatoms. According to Zhu et al. [91], by facilely varying the building blocks of CMPs, N-rich, metal-free electrocatalysts—based on hard carbon nanotubes derived from porphyrin-based CMP—exhibit excellent catalytic activity for an ORR in an alkaline medium. In particularly, the unique, hollow cylindrical geometry guarantees the sufficient exposure of the N-C active site, due to the designable flexibility and synthetic diversity of CMPs.

### 3.3. Defective Structure

Despite the heteroatom doping and microarchitecture engineering, the creation of defective structures likewise endows carbon nanomaterials with great potential in modulating their electronic structures and enhancing their electrocatalytic performance. According to their formation procedure, the defective structures in carbon nanomaterials can be classified into three types: topological defects, vacancy defects, and edge defects.

#### 3.3.1. Topological Defects

**Figure 10:**
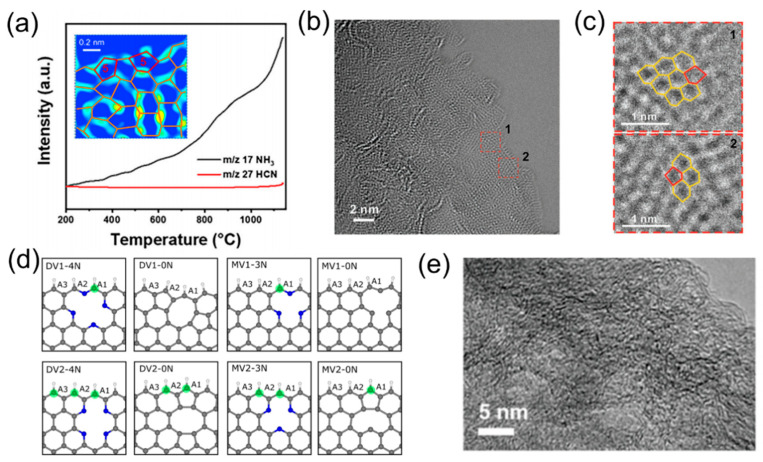
(**a**) Thermogravimetry mass-spectrometry (TG-MS) analysis of gases evolved from the thermal degradation of pyrrolic-N-dominated carbon monolith. Insets: the corresponding aberration-corrected atomic-resolution high-angle annular dark field scanning transmission electron microscopy (ACHAADF-STEM) image of D-cM. Reproduced with permission [94]. Copyright 2020, Elsevier. (**b**) The ACTEM image of abundant pentagon-defects. (**c**) Enlarged images from the red dash squares from areas 1 and 2 in (**b**). Reproduced with permission [95]. Copyright 2021, Wiley-VCH. (**d**) Investigation of the effect of defect conditions on oxygen adsorption, including the edge-closest, Py-N doping, divacant nanoribbon, monovacant nanoribbon, and non-doped nanoribbon. Reproduced with permission [97]. Copyright 2022, American Chemical Society. (**e**) TEM images of JCNT−0.5 with abundant edge defects. Reproduced with permission [106]. Copyright 2022, Elsevier.

A topological defect is generated by the distortion of the periodic carbon hexagons, which usually exist in the form of pentagon (C5-1), pentagon-octagon-pentagon (C5-8-5), pentagon-heptagon-heptagon-pentagon (C5-7-7-5), etc. Theoretically, the pentagon defects in the basal plane could modulate the local electronic structure and the contraction of band gap, leading to reduced Gibbs free energies for all the steps of ORR and enhanced ORR activity [92]. Inspired by this, Zhu et al. [93] developed a pentagon-defect-rich carbocatalyst through the in situ etching of fullerene molecules. Electrochemical measurements confirmed the pentagon defects indeed exhibited a 4e^−^ ORR mechanism, similar to commercial Pt/C, and a high E_1/2_ up to 0.833 V (vs. RHE). Using DFT calculations, Wang et al. [94] discovered that, through the removal of the specific nitrogen atoms, the corresponding topological defect structures could be created (Figure 10a). For example, removing graphitic-N sites leads to divacancy (C585); yet, the high formation energy of 7.35 eV indicates that such transformation requires more energy input. In contrast, the removal of pyridinic-N sites and pyrrolic-N sites is easier, which could lead to the separate pentagon (S-C5) and adjacent pentagons (A-C5), respectively, with a low formation energy below 2 eV. Inspired by this calculation result, they adopted a Zn-induced edge engineering strategy to synthesize three pristine carbon models by using phenol-formaldehyde nanofibers (PFNs) as the precursors. Structural analysis and electrochemical measurements indicated that the A-C5 defects presented the highest intrinsic activity for ORR, while C585 defects possessed the best performance in hydrogen evolution reaction (HER).

To tackle the problem of the inferior acidic ORR performance for carbon nanomaterials, Liu et al. [95] proposed that graphitic nitrogen (GN)-bonded pentagons in graphitic carbon could serve as a critical structure by DFT calculation. Experimentally, they synthesized the target catalytic sites, starting from the nitrogen-containing organic molecules, which were impregnated and pyrolyzed with metal halides (e.g., MgCl_2_, CaCl_2_, NaCl, etc.). Due to the effect of the halogen salts, abundant pentagon defects were successfully created in situ (Figure 10b,c), which enhanced the intrinsic activity of the carbocatalyst, combined with the further-exposed graphitic N active site, induced by the exfoliated graphene layers, and a high ORR performance (E_1/2_ = 0.81 V vs. RHE and much better stability) in an acidic medium. DFT calculation was further implemented to discern that the conjugated π−electrons at the edge catalytic site can be tuned by the GN-bonded pentagon defects, implying that the N-dopants, interacting with the topological defects, could contribute to the enhancement of acid ORR performance.

#### 3.3.2. Vacancy Defects

Vacancy defects arise due to the escape of carbon atoms in the hexagonal lattice of carbon. Based on the number of missing carbon atoms in the hexagonal lattice, vacancies can be classified as single vacancy defects (only one missing carbon atom), double vacancy defects (two missing carbon atoms), and multi-vacancy defects (more than three missing carbon atoms) [96].

Generally speaking, heteroatom evaporation is a common method employed to produce vacancy defects. Theoretically, Choi et al. [97] confirmed that vacancy defects could reduce the structural strain during the ORR, and thus lower the energy barrier for the adsorption of oxygen. Molecular dynamics simulations indicated that the pyridinic-N species was a universal method employed to create the vacancy defects for catalyzing the ORR process (Figure 10d). Qian et al. [98] developed a versatile platform to convert the sulfur-containing tubular polymer into diverse heteroatom doped porous carbon nanotubes through heat treatment. The evaporation of S, during the pyrolysis process under an ammonia atmosphere, creates numerous vacancy defects, which are demonstrated as active centers for the 4e^−^ ORR. It is worth mentioning that when the atmosphere for the pyrolysis process is changed to oxygen, the resultant O- and S-co-doped carbocatalyst shows excellent 2e^−^ ORR performance with a totally different reaction mechanism from that of the 4e^−^ ORR. By N_2_ plasma treatment, Wee et al. [99] developed nanosized holes on the sidewalls of single-walled carbon nanohorns (SWNHs). The abundant holes, decorated with the pyridinic-N sites, were recognized as the active sites for ORR. Although the physical [100] or chemical [101] etching approaches are widely adopted to create vacancy defects—including the ball milling, MgO templating, KOH, and NH_3_ activating—the resultant defective carbocatalysts are always uncontrollable in terms of their defect configurations and numbers.

In addition, the presence of M-Nx in a metallic POP could also be converted into vacancy defects if the M-Nx moieties could be converted into MOx. However, the complete formation of such a domain-confined etching vacancy, by the above conversion, may be impeded because of the high stability of the M-Nx moieties. Ye et al. [102] proposed a halide-mediated “bait-and-switch” mechanism to thermally convert the M-Nx moieties into the endogenous MOx, realizing the N/DC coupling sites in metal-free carbon with a stronger electron-donating capability. During this process, the vacancy defects were created by the endogenous ZnO, during the pyrolysis process, which could simultaneously construct abundant N/DC coupling sites for superior ORR performance with E_1/2_ = 0.903 V. Yan et al. [103] prepared a melamine–phytic acid supermolecular aggregate (MPSA) by using the melamine and phytic acid as the monomers, followed by a direct pyrolysis, with the help of Co^2+^, resulting in a N/P dual-doped carbocatalyst with enriched holes on the graphene-like surface. An excellent ORR performance in alkaline electrolyte was achieved, which was even close to the commercialized Pt/C catalyst.

#### 3.3.3. Edge Defects

When the escape of some carbon atoms occurs along a fixed direction, such as the zigzag or the armchair orientation, the defective structure is classified as an edge defect. Impressively, Yu et al. [104] designed 10 different polycyclic aromatic hydrocarbons (PAHs) with tunable carbon atom numbers, and precise edge configurations and gradient structures. In this case, PAHs with an exclusive armchair (e.g., phenanthrene [PT]) or zigzag configuration (e.g., anthracene [AT]) or the armchair–zigzag hybrid configuration (e.g., coronene [CN]), were successfully synthesized. They monitored the key intermediates, e.g., O_2_ (adsorption) and superoxide anion O_2_^−^* by the in situ time-resolved attenuated total reflectance infrared (ATR−IR) spectra and simulation calculations. Additionally, the structure–property relationships between the details of edges, and the ORR activities interpreted in both armchair and zigzag configurations are beneficial for the 2e^−^ ORR.

The sacrificing template method is a common strategy for the construction of edge defects. For example, silica and polystyrene (PS) microspheres could be utilized to introduce edge defects, but the subsequent removal of these templates requires harsh conditions, which will exacerbate environmental pollution. Chen et al. [105] explored a polyaniline (PANI) sphere with manganese oxide (MgO) spheres as the self-surfacing template, and the obtained nitrogen and phosphorus co-doped porous carbon spheres (NPCSs) featured abundant edge defects and efficiently exposed N/P sites, resulting in an excellent bifunctional properties for both ORR and OER.

The salt-assisted exfoliation of the POPs is also an effective method to create desirable defects. Based on the typical polyaniline polymers, Jin et al. [106] innovatively introduced the subsequent ZnCl_2_-assited carbonization and NH_3_-activation process, resulting in jagged carbon nanotubes (JCNTs) with abundant native zigzag or armchair edge defects (Figure 10e). The authors attributed the origin of the edges to the alternative connection between the phenyl ring and the amino groups. Combined with the DFT calculations, the edge defective structures were disclosed to be capable of inducing a large strain at the atomic level, activating oxygen, reducing the adsorption energy barrier, and facilitating the reaction kinetics for ORR. Xia et al. [107] exfoliated the elaborately synthesized ZIF nanoleaves (Zn-ZIF-L) by using metal chlorides (LiCl and KCl) as the exfoliators. The structural advantage of abundant nanopores and defect active sites conferred excellent ORR activity, in acidic electrolytes, to the derived carbocatalyst. Wang et al. [108] synthesized carbon rods consisting of defective nanosheets by carbonization of crystalline poly tannic acid (PTA) rods. Importantly, the spatially confined two-step localized contraction generated curly nanosheets with abundant edge defects during the heat treatment. The abundant edges, large surface area, and high porosity of the 3D sponge-like carbon superstructure synergistically confer it with superior electrocatalytic ORR performance, including an electron transfer number above 3.7, E_1/2_ = 0.78 V and a high current density of 1.34 mA cm^−2^. The authors attributed the high intrinsic activity to the high content of D1- and D2-type defects. Similarly, the direct pyrolysis of nitrogen-containing COP was demonstrated to be an efficient method to produce edge-rich porous carbon with a holey graphene-like structure [109]. In particular, the COP was built from 4-formylphenyl-substituted dichlorotriazine and phloroglucinol monomers with a hexagonal skeleton. The derived carbocatalyst, after thermal treatment, could inherit its holey nature and develop abundant pyridinic and graphitic-N sites, which exhibit a high limiting current density of 5.0 mA cm^−2^ for ORR.

In fact, the edge defects could also function as anchoring sites for the stabilization of heteroatoms. Chang et al. [110] discovered an edge-enriched N/S co-doped carbocatalyst by pyrolyzing a thiourea (TU)@ zeolitic imidazolate framework-8 (ZIF-8) composite. The abundant edges not only acted as the intrinsic electrocatalytic center, but also interacted with the heteroatoms to facilitate the mass transfer during the ORR process. Through a nano-cutting reaction, based on multi-walled carbon nanotubes (MWCNTs), our group revealed a positive correlation between the edging degree and the anchored heteroatom content [111]. The resultant edge-promoted N/S co-doping carbocatalyst exhibited a high E_1/2_ of 0.78 V (vs. RHE) and a high tolerance to the methanol crossover effect.

## 4. Structure—Property Relationship Constructed by POP-Derived Carbocatalysts

Porous organic polymers (POPs) are a promising precursor for the synthesis of carbon-based electrocatalysts, due to their high porosity and tunable properties. The electrocatalytic performance of POP-derived carbon materials for electrocatalytic ORR is highly dependent on their structural properties.

**Figure 11:**
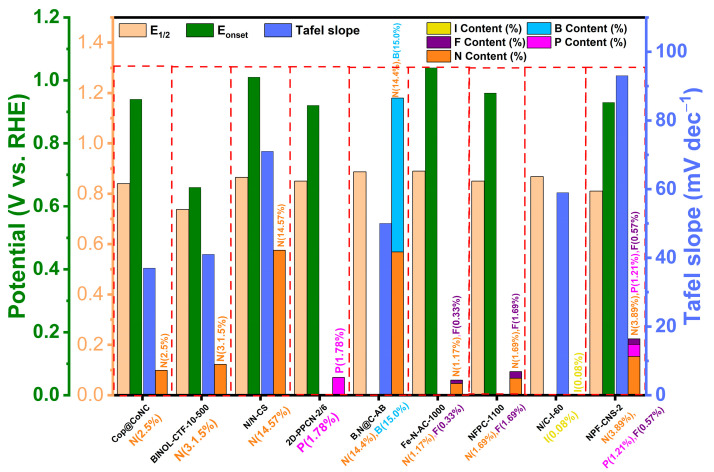
Relationship between heteroatom number/hybridization and ORR performance.

As described above, the catalytic activity of POP-derived carbon catalysts for the ORR is closely related to their heteroatom content (Table 1 and Figure 11), porosity (Table 2), and defective structure (Table 3). The introduction of heteroatoms, such as nitrogen, sulfur, and phosphorus, into the carbon structure can also enhance the ORR activity by modifying the electronic and chemical properties of the catalyst surface. Increasing the specific surface area and pore size of the catalysts can enhance their mass transport properties and increase their active sites for the ORR. Moreover, defects in the carbon lattice, often associated with heteroatom doping, can act as additional active sites, further promoting the ORR performance. These defects can also modulate the electronic structure of adjacent carbon atoms, potentially enhancing the catalytic activity by facilitating charge transfer during the ORR process. Herein, we make a summary for the recent progresses on the POP-derived carbocatalysts to correlate their specific structures and ORR properties.

In addition, the synthesis method and conditions used to prepare POP-derived carbon catalysts can also affect their ORR performance. Factors such as the type and concentration of activating agents, pyrolysis temperature, and duration can all influence the structural and chemical properties of the resulting carbon catalysts.

Overall, the structure–property relationship of POP-derived carbon catalysts for the ORR is complex and highly dependent on a variety of factors, and a delicate balance among these three structural factors—heteroatom content, porosity, and defects—is crucial in designing high-performance POP-derived carbon catalysts for ORR. By carefully tuning the structural and chemical properties of these materials, it is possible to achieve highly active and durable electrocatalysts for a range of energy conversion applications.

## 5. Conclusions and Outlook

The development and progress of POP-derived carbonaceous ORR catalysts offers a promising avenue for the design and synthesis of sustainable, efficient, and multifunctional electrocatalysts for energy storage and conversion. The design of POP-derived carbocatalysts, on the molecular level for high-performance ORR, continues to be an active area of research, with significant progress being made in recent years. Further deep research on developing desirable POP-derived catalysts is essential for their practical applications. Some critical perspectives for the future development of POP-derived carbocatalysts are highlighted below:

(1) Rational design of precursor materials: the design of precursor materials with tailored structures and functional groups can significantly influence the properties of the resulting carbocatalysts. Rational design of the precursor materials, based on computational simulations, for example, can help identify the best monomers and functional groups for enhanced ORR activity.

(2) Introduction of heteroatoms: the introduction of heteroatoms such as nitrogen, sulfur, and phosphorus into the carbon structure can modify the electronic and chemical properties of the catalyst surface, leading to enhanced ORR activity and stability. The design of these heteroatom-doped carbon catalysts with optimal dopant types, positions, and concentrations is a promising strategy for further improving their ORR performance.

(3) Controlled synthesis conditions: the synthesis conditions used to prepare POP-derived carbocatalysts can significantly affect their ORR performance. The use of controlled synthesis conditions such as pyrolysis temperature, reaction time, and precursor-to-activating agent ratio can help achieve the desired porosity, surface area, and heteroatom doping levels of the catalysts.

(4) Integration with other materials: integration of POP-derived carbon catalysts with other materials, such as metal nanoparticles, metal–organic frameworks, or graphene can improve their ORR performance through enhanced mass transport properties, increased active site density, and optimized electronic structure. The design of such composite materials, with optimal combinations of carbon and other materials, is a promising approach for further improving ORR activity.

(5) Advanced characterization techniques: advanced characterization techniques, such as in situ spectroscopy, electron microscopy, and X-ray diffraction, can provide detailed insights into the structure and behavior of POP-derived carbon catalysts under ORR conditions. The design of such advanced characterization techniques to elucidate the reaction mechanism and the formation of active species on the catalyst surface is an important area of research.

In summary, POP-derived carbocatalysts exhibit unique advantages for high-performance ORR, primarily owing to their distinctive active sites, identified and designed at the molecular level. These include heteroatoms, porosity/microarchitecture, and defective structures. Emphasizing the recognition, design, and increase in active site density, we have highlighted our unique perspective within this field of research. Future research in this area may focus on the combination of new strategies to achieve even higher ORR performance and to better understand the underlying reaction mechanisms. In addition to ORR, POP-derived carbonaceous catalysts could be engineered to perform other electrochemical reactions, such as oxygen evolution reaction (OER), hydrogen evolution reaction (HER), and carbon dioxide reduction reaction (CO_2_RR). This could result in the development of multifunctional electrocatalysts that can perform multiple reactions simultaneously, leading to more efficient and sustainable energy conversion.

## Figures and Tables

**Table 1:** Summary for the relationship between heteroatoms and ORR performance.

Name of POPs-Derived Carbon	Electrolyte	E_onset_/V (vs. RHE)	E_1/2_/V (vs. RHE)	Tafel Slope/mV dec^−1^	Heteroatom Content/%	Ref.
COP−Ppcfe	0.1 M HClO_4_	0.888	0.748	65.1	NA	[26]
Cop@CoNC	0.1 M KOH	0.94	0.84	37	~2.5 (N)	[27]
N/POPQ800	0.1 M KOH	0.832	0.728	NA	NA	[29]
COF-CN	0.1 M KOH	0.72	NA	61	NA	[30]
BINOL-CTF-10-500	0.1 M KOH	0.66	0.737	41.04	3.105 (N)	[34]
N/N-CS	0.1 M KOH	1.01	0.865	71	14.57 (N)	[35]
N,P−HCNF-8	0.1 M KOH	0.93	0.82	47	2.62–3.08 (N)	[38]
2D−PPCN-2/6	0.1 M KOH	0.92	0.85	NA	1.78 (P)	[39]
B,N@C−AB	0.1 M KOH	NA	0.887	50.2	14.4 (N); 15.0 (B)	[43]
Fe-N-AC-1000	0.1 M KOH	1.04	0.89	NA	1.17 (N); 0.33 (F)	[45]
NFPC-1100	0.1 M KOH	0.96	0.85	NA	0.68 (F); 1.69 (N)	[46]
N/C-I-60	0.1 M KOH	NA	0.868	59	0.08 (I)	[48]
NPF-CNS-2	0.1 M KOH	0.93	0.81	93	3.89 (N); 1.21 (P); 0.57 (F)	[51]

**Table 2:** Summary for the relationship between porosity and ORR performance.

Name of POP-Derived Carbon	Electrolyte	E_onset_/V (vs. RHE)	E_1/2_/V (vs. RHE)	Tafel Slope/mV dec^−1^	BET SSA/m^2^ g^−1^	Pore Volume/cm^3^ g^−1^	Pore Size Distribution/nm	Ref.
N-HCNF-2-1000	0.1 M KOH	1.01	0.84	111	368	NA	NA	[73]
NHCS-2	0.1 M KOH	1.002	0.893	77	918	1.18	27	[75]
GPCNSs	0.1 M KOH	0.958	0.897	48	1342	NA	0.7, 1.2 and 4.0	[76]
NHC/rGO-950	0.1 M KOH	0.95	0.83	74	1344	NA	4–10	[77]
NPF@CNF-800	0.1 M KOH	0.97	0.85	88	533.3	0.349	<1.0	[78]
NCF	0.1 M KOH	1.00	0.85	71	897.5	1.64	0.7, 1.5 and 25	[79]
Fe-SCNS	0.1 M KOH	0.99	0.89	NA	957	1.52	~20	[80]
CoFe20@CC	0.1 M KOH	1.02	0.86	58.8	342	0.438	NA	[81]
Co-N/S-DSHCN-3.5	0.1 M KOH	0.989	0.878	67	429	0.4	2–100	[82]
PA@TAPT-DHTA-COF 1000NH3	0.1 M KOH	0.957	0.847	110	1160	0.59	0.5–6	[86]
CC-3	0.1 M KOH	~0.90	0.828	101	436	NA	~2.5	[87]
800-N, P-CNT	0.1 M KOH	~0.87	0.805	NA	181.9	1.26	16.43	[88]
PYTA-TPEDH-COF	0.1 M KOH	0.69	NA	70	598	0.51	0.86	[90]
TPP-CMP−900	0.1 M KOH	0.95	0.83	NA	624	0.36	1.93	[91]

**Table 3:** Summary for the relationship between defects and ORR performance.

Name of POP-Derived Carbon	Electrolyte	E_onset_/V (vs. RHE)	E_1/2_/V (vs. RHE)	Tafel Slope/mV dec^−1^	Defect Type	Ref.
PD-C	0.1 M KOH	~0.87	0.78	NA	C_5_	[93]
D-CM	0.1 M KOH	~0.90	0.81	74	A-C_5_	[94]
S-1-900	0.1 M HClO_4_	~0.92	0.81	NA	C_5_	[95]
N-O-SWNH	0.1 M KOH	0.91	~0.80	NA	holes defects	[99]
N-hG6	0.1 M KOH	0.91	0.833	78	N-doping on the edge of holes	[101]
N/C-Br0.3	0.1 M KOH	~0.95	0.903	57	C_5_	[102]
JCNT-0.5	0.1 M KOH	~0.95	0.88	61	zig-zag/arm-chair edge	[106]
PTA-1000	0.1 M KOH	~0.90	0.78	74.2	edge defects	[108]
COF800	0.1 M KOH	0.86	0.79	NA	edge defects	[109]
NCF	0.1 M KOH	1.0	0.85	71	edge defects	[79]
NSCNT-6	0.1 M KOH	0.92	0.78	NA	edge defects	[111]

## Data Availability

Not applicable.
